# Multimodality imaging of simultaneous occurrence of cardiac transthyretin amyloidosis and cardiac sarcoidosis

**DOI:** 10.1007/s12350-022-03083-5

**Published:** 2022-08-16

**Authors:** Elena Elchinova, Yara Banz, Stephan Zbinden, Federico Caobelli, Christoph Gräni

**Affiliations:** 1 Department of Cardiology, Inselspital, Bern University Hospital, University of Bern, Freiburgstrasse 4, 3010 Bern, Switzerland; 2 Institute of Pathology, University of Bern, Murtenstrasse 31, 3008 Bern, Switzerland; 3 Regional Hospital Emmental, Cardiology, 3550 Langnau in Bern, Switzerland; 4 Department of Nuclear Medicine, Inselspital Bern, Bern University Hospital, University of Bern, 3010 Bern, Switzerland

## Introduction

We illustrate the findings of multimodality imaging (i.e., DPD-scintigraphy, 18F-fluorodeoxyglucose (18F-FDG) positron emission tomography (PET), cardiac magnetic resonance (CMR)) in endomyocardial biopsy proofed cardiac sarcoidosis and cardiac transthyretin amyloidosis (ATTR).

## Case presentation

A 79-year-old female presented with fatigue, dyspnea, elevated NT-proBNP, and recurrent exudative pleural effusion of unknown origin without bacteria or neoplastic cells. Pulmonary embolism, pneumonia, and tumor were ruled out by computed tomography. Echocardiography showed slightly reduced left ventricular ejection fraction with diffuse hypokinesia and apical sparing. Light-chain amyloidosis was ruled out and DPD-scintigraphy showed cardiac ATTR. As diagnostic thoracoscopy revealed no malignancy, but non-necrotizing granulomas in the lung, a FDG-PET was performed, which suggested multiorgan sarcoidosis. CMR showed diffuse fibrosis and multifocal late gadolinium enhancement (LGE) suggesting cardiac ATTR. However, the extensive LGE and multifocal inflammation would also be in accordance with cardiac sarcoidosis. Endomyocardial biopsy was performed, and histological analysis confirmed the diagnosis of both cardiac sarcoidosis and cardiac ATTR (differentiation revealed wild-type ATTR). Tafamidis and immunosuppressant therapy were initiated.

## Discussion

**Figure 1:**
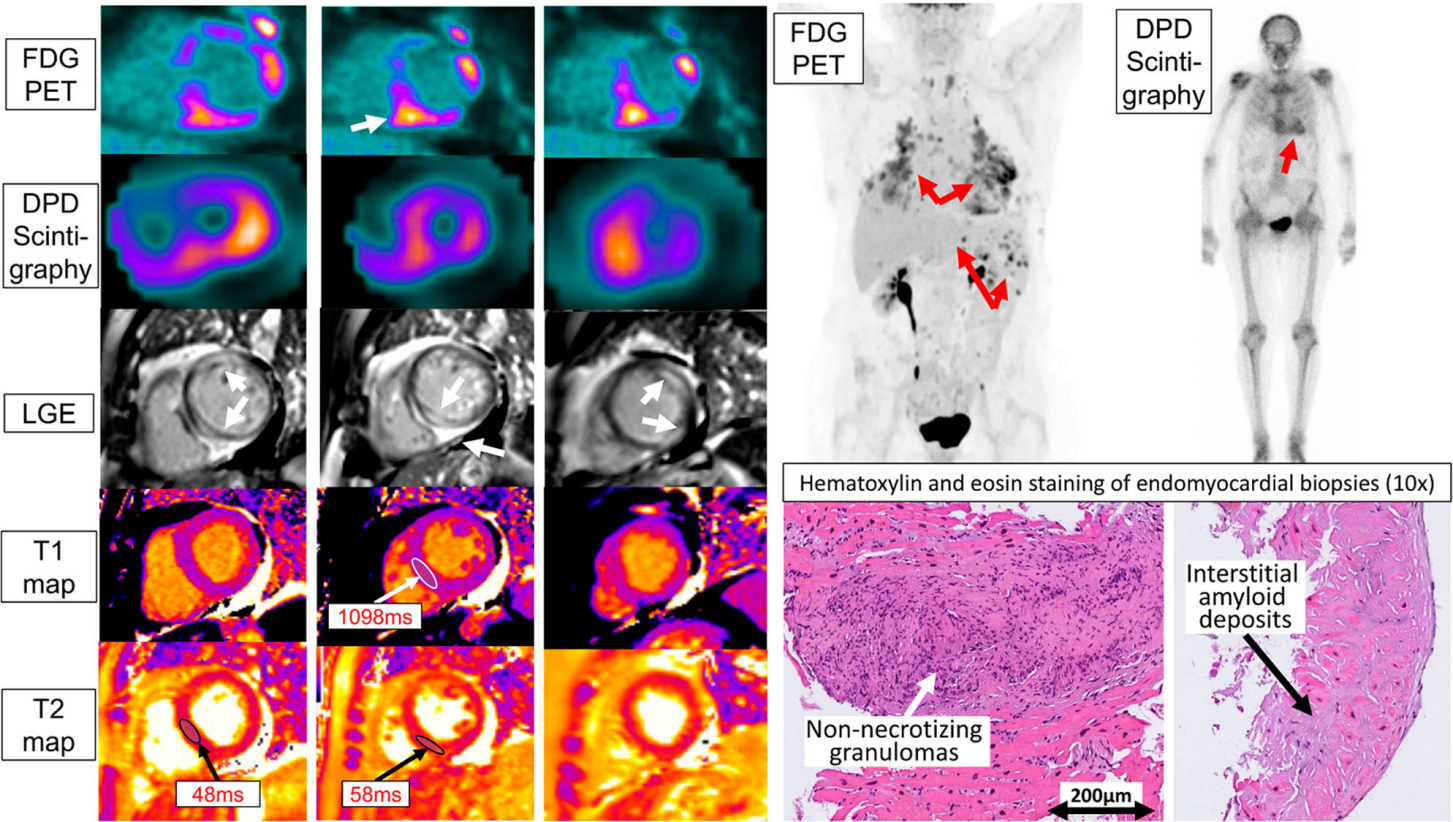
The cardiac short axis PET view on the upper left side shows multifocal FDG cardiac uptake. Multiorgan FDG uptake is depicted on the upper right side. DPD-scintigraphy showed Perugini grade 2 consistent with cardiac ATTR. LGE shows extensive scarring in in the endocardium, epicardium, midmyocardium, and diffuse fibrosis with elevated T1 mapping values and extracellular volume fraction (between 42 and 51%) were measured throughout the myocardium. T2 mapping shows multifocal edema in the myocardium. Hematoxylin, eosin, and congo red staining of endomyocardial biopsy confirmed simultaneous occurrence of cardiac sarcoidosis and cardiac amyloidosis (further differentiation revealed wild-type ATTR)

The main differentials according to the imaging findings were (a) FDG-PET uptake in LV-myocardium: cardiac ATTR mimicking cardiac sarcoidosis?^1^ (b) DPD-scintigraphy uptake in LV-myocardium: cardiac sarcoidosis mimicking cardiac ATTR?^2^ (c) Simultaneous presence of both entities, which, to our knowledge, has not yet been described in the literature. Multimodality imaging and endomyocardial biopsy could establish the diagnosis of the simultaneous occurrence of cardiac ATTR and cardiac involvement of systemic sarcoidosis and helped to guide optimal medical therapy of this patient (Figure 1).
